# Retinal and Brain Microglia in Multiple Sclerosis and Neurodegeneration

**DOI:** 10.3390/cells10061507

**Published:** 2021-06-15

**Authors:** Soyoung Choi, Li Guo, Maria Francesca Cordeiro

**Affiliations:** 1 UCL Institute of Ophthalmology, London EC1V 9EL, UK; skgthoi@ucl.ac.uk (S.C.); l.guo@ucl.ac.uk (L.G.); 2 ICORG, Imperial College London, London NW1 5QH, UK

**Keywords:** retina, microglia, neurodegeneration, multiple sclerosis, retinal microglia, microglia morphotype

## Abstract

Microglia are the resident immune cells of the central nervous system (CNS), including the retina. Similar to brain microglia, retinal microglia are responsible for retinal surveillance, rapidly responding to changes in the environment by altering morphotype and function. Microglia become activated in inflammatory responses in neurodegenerative diseases, including multiple sclerosis (MS). When activated by stress stimuli, retinal microglia change their morphology and activity, with either beneficial or harmful consequences. In this review, we describe characteristics of CNS microglia, including those in the retina, with a focus on their morphology, activation states and function in health, ageing, MS and other neurodegenerative diseases such as Alzheimer’s disease, Parkinson’s disease, glaucoma and retinitis pigmentosa, to highlight their activity in disease. We also discuss contradictory findings in the literature and the potential ways of reducing inconsistencies in future by using standardised methodology, e.g., automated algorithms, to enable a more comprehensive understanding of this exciting area of research.

## 1. Introduction

Microglia are resident immune glial cells of the central nervous system (CNS). They dynamically shift into various different morphologies, which have also been associated with specific activation states that may be related to neuroprotective and/or neurotoxic functions in response to stimuli, injury or insult [1,2,3,4,5,6]. These morphological and functional changes are essential to support a healthy CNS by contributing to homeostasis [2,7,8]. However, emerging evidence has started to show the involvement of microglia in disease whereby microglial dysfunction may be caused by disease and/or actually cause augmented disease-associated pathologies [2]. Nevertheless, the exact degree of involvement and mechanisms of how microglia may influence health and disease is unknown and currently being investigated. The purpose of this review was to compile information on the characteristics of CNS microglia, including those in the retina, with a focus on their activation states, morphology and function, in relation to health and disease. In the first section, we have compiled an extensive description of the main features of microglia in the general CNS and in the retina. We also explain some of the microglial changes that occur throughout normal development and the ageing process. In the next section, we discuss the current understanding of multiple sclerosis (MS) as an autoimmune disease, the surprisingly common ocular manifestations of MS, microglia in MS and finally, microglia in other neurodegenerative diseases. Finally, we provide insight as to why there may be contradicting findings in relation to the characteristics of CNS and retinal microglia. Here, we suggest different methods of experimentation such as using automated algorithms in light of producing more conclusive and consistent results.

## 2. Microglia

The CNS is made of several different types of cells, 5–10% of which are microglia, the resident immune cells [9]. Microglia were originally thought to exist as quiescent or “resting” cells that continuously survey their microenvironment for any stimuli or injury that may be harmful [5]. Conversely, more recent findings have suggested explanations for their dynamic properties. Microglia can shift into different morphological states, which will be referred to as morphotypes. Each morphotype has been correlated to different activation states, which have also been associated with unique functions required to preserve a physiologically “normal” environment [5].

Once microglia are activated from their resting state, they can further differentiate into two main phenotypes: M1 and M2 [6]. Although there is a lack of understanding of the specific mechanisms that induce this differentiation, M1 and M2 microglia have also been associated with distinct cytokines, chemokines and trophic factors [6]. Pro-inflammatory responses are associated with the “classically” activated M1 microglia, which encourage neuroinflammation as a response to insult or injury, creating a neuro-toxic environment and removing dysfunctional fragments of cellular debris [6]. This may occur as a result of inflammatory factors such as interleukin-1ß (IL-1ß), tumour necrosis factor-alpha (TNF-alpha) and inducible nitric oxide synthase (iNOS) [6,10]. Conversely, the “alternatively” activated M2 microglia are notoriously responsible for anti-inflammatory responses that encourage neuroprotective and restorative processes [6,10]. More recently, at least three more sub-phenotypes of the M2 type (M2a-c) have been found [11] whereby, more specifically, the M2a type secrete anti-inflammatory factors such as IL-10 and insulin-such as growth factor-1 (IGF-1), promoting cell debris removal and neuroprotection [6,11,12]. The M2b is said to be stimulated by inflammatory factors such as IL-1ß and lipopolysaccharides (LPS), which may also increase the expression of IL-10 [11]. These M2b microglia have been found to have phagocytic properties in brains modelled for Alzheimer’s disease (AD) and expressing high levels of CD64 [11]. The M2c “acquire deactivation” by IL-10 or glucocorticoids, in turn increasing expression of growth factors such as TGFß [11]. Despite these differences, M1 and M2 activation are functionally required to guarantee the removal of dysfunctional cells or noxious aggregates of cellular debris [6]. M1 microglia are usually involved in the clearance of cell debris, and this inflammatory response must be controlled by M2 microglia to avoid needlessly prolonged inflammation [6]. Often in pathological processes, the typical balance of M1 and M2 polarisation seen in normal conditions may be affected [6]. This can result in the clearance of healthy cells due to excessive M1 inflammation and the M2′s dampening effect of M1s may be overwhelmed causing further damage [6]. This often occurs in neurodegenerative illnesses and therefore, some therapeutic candidates that target M1 and M2 polarisation have been proposed [13]. Despite this, there is also emerging evidence that suggests that the M1/M2 polarisation may be outdated. It was first introduced and used to accommodate easier methods of data interpretation [14]. However, recent advances in technology have revealed overlapping morphological and genetic characteristics between M1/M2 types, suggesting a need to re-evaluate microglia types [14]. More recently, disease-associated microglia (DAM) have also been recognised as a unique microglia type seen in disease [14]. DAMs are characterised by microglia that express low levels of surveillant and homeostatic genes and high levels of markers associated with degeneration such as triggering receptor expressed on myeloid cells 2 (TREM2) [14,15].

**Figure 1:**
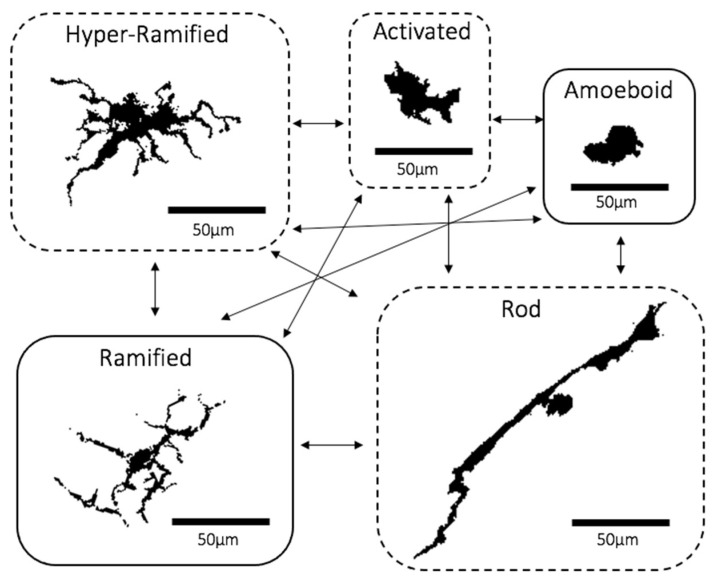
Microglia Morphotypes. A diagram showing microglia morphotypes with arrows showing potential orders of morphotype modifications. Dashed boxes represent the “transitioning” microglia. Microglia are from whole-mount retinal images of iba-1-stained C57BL6 mouse (28 months old) retinal microglia. Scale bar = 50 μm. Images were obtained from the Cordeiro Laboratory following similar protocols mentioned in Davis et al. [24].

With regards to morphology, there about five main microglial morphotypes that have been recognised, including the ramified, hyper-ramified, activated, amoeboid and rod types. Under non-primed or “inactive” conditions, microglia appear “ramified”. They are distributed evenly like a “mosaic”, with each consisting of a small and round cell body to which are attached several thin and long processes that constantly extend and retract to facilitate their surveillant functions (Figure 1) [2,5,16]. Experimental in vivo imaging of the brain has shown that these dynamic processes come into close proximity with neurons, glia and blood vessels, suggesting that microglia actively co-operate with other parts of the cortex to sustain a physiologically normal CNS environment [17]. Sometimes, ramified microglia may recognise microenvironmental changes and respond by changing into “hyper-ramified” microglia typically defined by more abundant processes that are longer and thicker, attached to larger, lobular and irregularly shaped cell bodies (Figure 1) [16,18]. Most types of primed “non-ramified” microglia, including hyper-ramified cells, are scattered in the CNS in an irregular and “clustered” distribution [2]. Hyper-ramified microglia may also shift into the activated form upon exposure to significantly noxious stimuli, which also have similar cell bodies to hyper-ramified cells whilst having much fewer processes that are thicker and shorter (Figure 1) [2,16,19]. When noxious stimuli are extensively prolonged, activated microglia may morph into the “amoeboid” state with a rounder, larger and more regularly shaped cell and very few or no processes (Figure 1) [16,18]. A recently re-discovered morphotype are “rod” microglia characterised by a long, sausage-shaped cell body with a few processes that may not always extend beyond the length of ramified microglia (Figure 1) [20,21]. Although emerging evidence has shown rod microglia localising near neurons and aligning itself along the nerve fibres, their exact function is still yet to be discovered [20,21,22,23]. The final type is the amoeboid microglia, often referred to as the phagocytic type that moves to the site of damage and phagocytose dead or dying neurons and cell debris (Figure 1) [5,24]. Recently emerging evidence has led to a theory that the hyper-ramified, activated and rod morphotypes may be “transitioning” forms that exist between the ramified and amoeboid states [5,25].

### 2.1. Retinal Microglia

**Figure 2:**
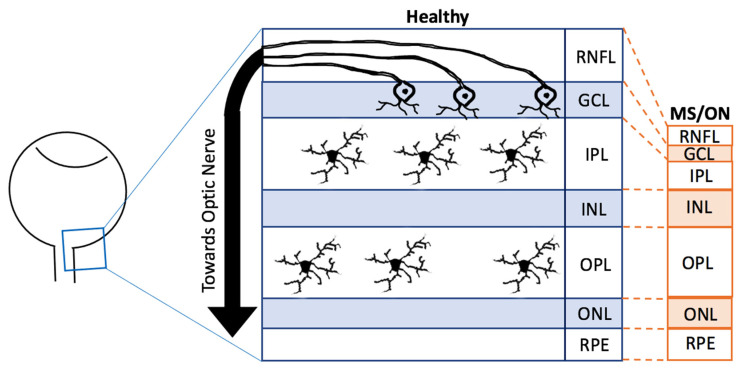
Comparison of retinal changes in Healthy and Diseased Retinas. A schematic diagram showing changes in the main layers in the healthy (blue) and MS/ON (multiple sclerosis/optic neuritis, orange) retinae. The diagram is not to scale and a hypothetical representation. The ganglion cell axons are found in the RNFL whilst their cell bodies are in the GCL. Microglia are said to reside in the IPL and OPL. There are also other retinal neurons and glia that are not specified in this diagram. RNFL = retinal nerve fibre layer, GCL = ganglion cell layer, IPL = inner plexiform layer, INL = inner nuclear layer, OPL = outer plexiform layer, ONL = outer nuclear layer, RPE = retinal pigment epithelium, MS = multiple sclerosis, ON = optic neuritis.

The retina is an essential part of the CNS. Due to its transparent nature to light, it is possible to use less invasive and high-resolution imaging modalities to visualise the retina [26,27]. It is predominantly known for its involvement in converting light energy into electrical signals [28]. In humans, the retina develops from the first month embryologically to the end of the first year, originating from the neuroectoderm [28,29]. The retina consists of several distinct layers with the innermost layer being the retinal nerve fibre layer (RNFL), then the ganglion cell layer (GCL), the inner plexiform layer (IPL), the inner nuclear layer (INL), the outer plexiform layer (OPL), the outer nuclear layer (ONL) and the final layer the retinal pigment epithelium (RPE) (Figure 2) [29]. Throughout these layers exist several types of cells, including amacrine cells, Müller cells, astrocytes, horizontal cells, rod and cone photoreceptors and bipolar cells, all of which may also be found in the rest of the CNS [29].

Approximately 0.2% of the total retinal cells are made of microglia, of which 50% usually reside in the IPL whilst the rest reside in the OPL (Figure 2) [2]. Through development and homeostasis, microglia dynamically move through the different layers of the retina, although avoiding the ONL [2]. Furthermore, many retinal diseases and retinal injury models have shown that microglia may migrate towards the region of degeneration, become activated and proliferate [30,31], as described in more detail later. Although both retinal and brain microglia are developed from the primitive yolk sac, the appearance of each morphotype may vary depending on the region of the CNS [10]. For example, microglia in the striatum, hippocampus and frontal cortex have larger cell bodies with more processes compared to those in the cerebellum [32]. Additionally, there is evidence that distinct layers of the cerebral cortex contain microglia of different sizes [32]. Morphotype appearances may also vary depending on the methods used or the axes of dissection. For instance, ramified microglia may appear horizontally ramified, which, in a cross-sectional observation, appear as one horizontally long cell whilst in a whole-mount observation, appear as and like the previously mentioned standard morphological description of ramified microglia [2,33]. When microglia are primed and become hyper-ramified, their processes extend radially, reaching across different layers, which is more visualisable in cross-sectional dissection compared to whole-mount observations [2,33]. Despite these differences, retinal and brain microglia have been found to share the expression of several transcription factors [2]. Numerous studies have also been able to observe each morphotype in both the brain and the retina [2,4,5,22,33,34]. However, it is still not clear whether or not each morphotype in both CNS regions share the same features [2].

### 2.2. Molecular Markers and Stimuli Affecting Microglial Morphology

Similar to the brain, individual microglia morphotypes in the retina may result from microglial responses to various cytokines, chemokines or damage-associated molecular patterns present in its microenvironment, suggesting heterogeneity in microglial genetic expression [9]. For instance, ramified microglia are said to have high expressions of P2RY12, which is often associated with surveillant functions, whilst amoeboid microglia have been found to express high levels of CD68—a notorious marker of phagocytosis [35,36]. Differential expression levels of microglial ion channels and surface receptors can also interact with such molecules (e.g., cytokines) in the microglial microenvironment, resulting in microglial changes including density, spatial distribution, activation state and morphotype and disease pathogenesis [9,37]. For instance, transforming growth factor beta (TGFß) is an important microglial cytokine, pleiotropically involved in the physiological development of retinal neurons and vessels [37]. Ma et al., observed iba-1 positive microglia, from whole-mounted retinas of tamoxifen-induced ablation of TGFßR2 (TGFß receptor) in 2-month-old Cx3cr1^CreER/+^, Tgfbr2^flox/flox^ mice [37]. The microglial morphology appeared ramified at 1 day post TGFßR2 ablation (PTA), which then became less ramified with “stubby” processes by 2–5 days PTA and finally appeared with lengthy processes that were aligned along the retinal blood vessels by 3–10 weeks PTA [37]. Real-time polymerase chain reaction (RT-PCR) analyses of TGFßR2 ablated retinal microglia revealed decreased expression of growth factors (e.g., BDNF, PDGFA) and increased expression of inflammatory activation markers (e.g., MHCII, CD68). This was not observable in healthy microglia. Additionally, whilst the TGFßR2 ablated animals showed no effects of the retinal vasculature, they experienced retinal thinning and amplified rates of pathological choroidal neovascularisation in response to injury [37].

### 2.3. Extracellular Vesicles: Effects on Microglia

Retinal (and brain) microglia also secrete extracellular vesicles (EVs), which are membrane-bound particles composed of mRNA, miRNA, DNA, cytokines, lipids and proteins, regarded as a biopsy of its origin cell [6,38,39]. EVs are involved in cell-to-cell communication by transporting their neuro-protective/toxic components in response to intracellular and extracellular cues as they move through the bloodstream and cerebrospinal fluid (CSF) to reach other cells within close and distant proximity [6,38,39]. EVs include exosomes and ectosomes, which consist of apoptotic bodies and microvesicles [39]. Firstly, many endosomal vesicular bodies fuse to form exosomes (40–160 nm in size) which are then released for intercellular communication [39]. Secondly, microvesicles instead originate from the plasma membrane undergoing outward budding (100–1000 nm) [38]. Finally, apoptotic bodies are >1000 nm in size and form through membrane blebbing of disintegrating, e.g., retinal microglia [38]. In fact, components of apoptotic bodies such as phosphatidyl serine modulate microglial phagocytosis [38,39]. As a result of these diverse features, microglial EVs have recently started to be investigated in relation to neurodegenerative diseases.

### 2.4. Microglia and Ageing

Microglial cells in the retina of newborn and postnatal rats have a round or amoeboid form and show pseudopodal processes involved in cell debris phagocytosis and developmental synapse remodelling [2]. Steadily, as the second and third week of the postnatal period approaches, microglial cell bodies become smaller with fine ramifications [2], assuming a highly ramified phenotype with the progress in brain development [40]. This progression is also reflected in brain regions, including the cerebellum, which suggests that microglia are actively involved through the maturation of the CNS.

A study investigated cortical microglia characteristics in old adult (24 months) and young adult mice (6 months) to find decreased microglial density and irregularly distributed clusters [41]. However, retinal microglia studies have revealed some contradictory results. Firstly, similar age groups of mice as the previous study were examined (18–24 months vs. 3–4 months) to find that the older adults had significantly elevated retinal microglia densities compared to that of younger adult mice [42]. This suggests that the microglial responses to ageing may be region-specific. Further inspection of real-time retinal imaging showed that most of the old adult microglia appeared to have fewer and shorter processes, suggestive of activated or amoeboid microglia, than those in the young mice [42]. These were found to be arranged in a mosaic distribution. A more recent study looked at the morphological and marker expression differences of whole-mounted retinal microglia in old (15 months) and young (age not specified) mice [43]. Unlike the results from Damani et al., this study found no significant differences in iba-1 positive retinal microglia cell density and area covered by the processes between the two age groups, whilst the cell soma area in the OPL, IPL, NFL and GCL and the number of vertical processes had significantly increased [42,43]. Additionally, the young microglia cells were mostly expressing P2RY12 and no CD68, whilst the aged microglia were CD68+ and appeared amoeboid [43]. These results, instead, imply that with ageing, microglia may shift their morphologies and genetic profile to accommodate the ageing process [43]. Fernández-Albarral et al., however, do not disclose the exact ages, species of mice or the quantitation methods used. Such differences between studies may impact the results.

Aged microglia become less dynamic, showing significantly slower process motilities compared to those in their younger counterparts, which likely compromise their ability to continuously survey and interact with their environment [42]. Post-mortem hippocampal and cortical investigations in “young”, “middle”, or “old” (20–69 vs. 70+ vs. 90+ years old) adults revealed more region-specific microglial responses to ageing [4]. Rod-shaped microglia were found to be significantly more abundant in the “middle” compared to “young” adults, although this was only seen in the hippocampus whilst the “old” adults only had significant rod increases in the hippocampus [4]. Other cortical and hippocampal studies found similar microglial trends with age, which suggests that aged microglia may have diminished surveillant capabilities required for maintaining a healthy CNS, which in turn may increase the risk of developing neurodegenerative diseases [44,45,46].

Myelinated axons of neurons are an essential feature of the CNS that enables efficient action potential conductance [47]. Whilst myelin is produced by many oligodendrocyte cells, it undergoes constant renewal, with myelin debris being directly removed and indirectly replaced by microglia [47,48]. However, it has been proposed that changes in myelin debris formation with ageing may also result in age-related dysfunction of CNS immune cells such as microglia [49]. Hence with age, there are more impaired myelin-associated molecules, and a higher myelin protein turnover rate is required [49]. This leads to an increased rate of myelin breakdown, causing burdensome myelin accumulation, which then forms insoluble lysosomal aggregates within microglia cells [49]. TREM2 are expressed on microglial cell surface membranes; however, its deficiency or mutation can result in disease caused by excessive demyelination [50]. Poliani et al. found “aged” TREM2 deficient brains had demyelination with dystrophic and amoeboid looking microglia [50]. It is also said that TREM2 positive amoeboid microglia may then morph into different shapes as it starts to produce factors such as TNF and IL-1, which are considered to be “pro-regenerative” factors [48]. Thus, the extracellular matrix is modified to trigger modifying the extracellular matrix to attract and activate oligodendrocyte precursor cells (OPCs) which then remyelinate the axon [48]. Furthermore, healthy microglial responses to demyelination were found to increase expression of genes associated with activation, phagocytosis and lipid metabolism, whilst that of TREM2 deficient microglia were found not to [50]. Other studies have shown that although debris clearance by microglial phagocytosis is greater with the increase in age, “younger” myelin phagocytosis was more proficient than with “older” myelin [51]. These age-related changes were also correlated with the more frequent appearance of “dystrophic” non-ramified microglia [51]. There are not yet many investigations of myelin debris related retinal microglial phagocytosis, possibly due to the lack of myelin in the retina itself.

With ageing, the microglial populations become dystrophic and undergo structural and morphological changes. The cytoplasm starts to fragment, their cell processes gradually lose the fine ramifications and show spheroidal swellings [52]. In addition, their constitutive microglial function starts to decline and show abnormal microglial injury responses. These alterations, combined with molecular and gene expression ageing changes within microglia, resulting in their reduced capability of maintaining homeostasis in the immune environment, and this may contribute to neuronal impairments, cognitive decline and age-related diseases [5,27,53,54,55].

### 2.5. Genetic Factors: Effects on Ageing Microglia

Age is a well-known risk factor for neurodegenerative diseases [56,57,58]. Therefore, the change of gene expression in the ageing retina has become of significant interest. For instance, Chen et al. investigated the total retinal RNA of 3-month- and 20-month-old C57BL/6 mice [57]. With the increase in age, 298 genes, including those related to stress response and glycoprotein synthesis, were upregulated more than two-fold whilst 137 genes, including those related to immune and defence responses, had also been downregulated more than two-fold [57]. Additionally, RT-PCR analyses showed increases in inflammatory cytokine-, chemokine-, or complement activation-associated genes, e.g., chemokine (C-C motif) ligand 2 (CCL2), CCL12 or complement component 3 (C3) [57]. The authors then hypothesised that this might reflect microglial activation, which was supported by the immunohistological observation of isolectin B4^+^ amoeboid microglia in the IPL of aged mice only [57]. Another study was able to specifically investigate transcriptional changes in the ageing retinal microglia by comparing the RNA extracted from isolated retinal microglia of 3-, 12-, 18-, and 24-month-old C57BL/6 mice [58]. A total of 719 differentially expressed genes were identified and were functionally associated with microglial immune regulation, e.g., IL3 and IL7, angiogenesis, e.g., vascular endothelial growth factors and trophic growth factors, e.g., neurotrophin [58]. Interestingly, like Chen et al., expression of C3, a gene associated with age-related macular degeneration (AMD), increased with age which implied that senescence-associated retinal microglia transcriptional changes might contribute to AMD pathogenesis [58].

## 3. Multiple Sclerosis

### 3.1. Multiple Sclerosis

Multiple Sclerosis (MS) is a chronic inflammatory disease of the CNS, affecting approximately 100,000 patients in the U.K. and 2,500,000 patients globally [59]. Interestingly, it can affect up to three times as many women as men, with an average age of onset in early adulthood [60]. Whilst the initial records of pathophysiological features related to MS date back to 1838, their pathological and clinical presentations were first identified as “MS” in 1863 by Jean-Martin Charcot [61]. Since then, the complex pathologies of MS have been revealed. Frequently occurring symptoms of MS involves the ocular pathway such as RNFL thinning, optic neuritis (ON) characterised by inflammatory damage to the optic nerve and uveitis characterised by intraocular inflammation of the vitreous body, retina, and uveal tract [62,63,64,65,66]. It has also been reported that such retinal changes may occur before changes in the rest of the CNS in many neurodegenerative diseases, including MS [26]. These recent findings have led to much interest in retinal research. Despite the complex processes involved in MS development, it is a disease defined by autoimmune responses through activation of immune cells such as T-lymphocytes, B-lymphocytes, microglia cells and macrophages, demyelination, remyelination and neurodegeneration [12,67,68,69].

A well-recognised hypothesis of MS is that it develops through two main phases. Firstly, T-cells and B-cells mediate inflammatory responses by releasing cytokines which induce activation of inflammatory cells such as microglia behind a “closed” blood-brain barrier (BBB) [68,70,71,72]. Chronic inflammation can then result in mitochondrial dysfunction, causing energy deficiency. Secondly, the neuroprotective signals are over-ridden, which impairs the ability to repair demyelination, damaged axons and neurodegeneration [68,70]. As a result, there are significant blockages of axonal conductance whereby eventually, the patient is left with irreversible lesions of the CNS such as the BBB, causing it to become “leaky” [68]. This, however, remains a theory due to recent emerging evidence showing that some patients respond better to therapeutics agents that target B-cells as opposed to those that target T-cells [70]. Although there have been multiple investigations using ever-evolving methods, there is a lack of consensus due to contradictory scientific results [73].

### 3.2. Sub-Types of Multiple Sclerosis

There is a spectrum of severity for symptoms and rates of progression, reflecting the heterogeneity of MS [70]. The earliest presentation of MS patients can be recognised as a clinically isolated syndrome (CIS) [67]. CIS patients have monophasic and monofocal symptomatic episodes, experiencing, e.g., ataxia, photophobia or areflexia, which lasts between 24 h at 3 weeks [67]. Primary progressive MS (PPMS) affects around 15% of MS patients who usually experience a steady and progressive decline in health [74]. Progressive relapsing MS (PRMS) is the least common form of MS, which only affects around 5% of patients that experience a steady decline in health with unexpected spikes of deterioration and recovery [74]. Relapse remitting MS (RRMS) is a more common form, affecting 80–90% of patients that experience unforeseen surges of disability [70]. Usually, these surges are succeeded by complete recoveries, but as patients age and progress to later stages, these recoveries become more partial [70,74]. This may be justified by many RRMS patients proceeding on to develop secondary progressive MS (SPMS), where patients may experience fewer spikes of impairment and start to mimic the disease trajectory of PPMS [70,74]. Recently, wider interests in genetic studies have enabled MS-specific alleles and gene variants to be recognised, especially through the genome-wide association study [70,75]. Large amounts of data have accommodated more sophisticated analyses that revealed that specific gene variants were correlated to the various types of MS [76]. These gene variants were predominantly found physically and functionally near to immunomodulatory genes associated with MS pathogenesis [70,75].

Despite this, the profiles of these sub-types are only descriptive as there is a lack of sufficient evidence to make accurate pathophysiological distinctions [77]. For instance, a large proportion of MS patients experience asymptomatic phases whereby MS-associated lesions and other pathophysiological changes may occur silently [78]. Some post-mortem brain studies have even shown that there were distinctive MS-associated pathologies seen in subjects who had been considered “healthy” during their lifetime [78]. More recently, references to these sub-types have therefore evolved, distinguishing between disease activity or no disease activity with details of “surges” or steady progression and observations of new CNS lesions [77].

### 3.3. Ocular Manifestations of Multiple Sclerosis

Post-mortem and retinal imaging studies have revealed MS-related pathologies such as optic nerve damage by demyelination and atrophy and GCL, IPL, and RNFL thinning (Figure 2) where the RGCs and microglia can be found [79,80]. It is theorised that retinal thinning is a downstream effect of the optic nerve and RGC degeneration following microglia-mediated immune responses [79,81]. Interestingly, these hallmarks are symptomatically present in more than 20% of pMS and asymptomatically present in more than 68% of pMS, making almost a staggering 90% of pMS who are estimated to have such retinal pathologies. The typical symptomatic features consist of photopsia, monocular loss of visual acuity and colour vision with mild eye pain, whilst some atypical features consist of photophobia, binocular prolonged vision loss and severe or absence of pain [62,67,82]. These symptoms may develop from a couple of hours to days, peaking at roughly 2–3 weeks from the initial signs followed by recovery [83].

Clinicians have been encouraged to use neuroimaging techniques such as magnetic resonance imaging (MRI) to confirm MS-associated diagnoses [63,79]. Such neuroimaging methods can be used to image the entire CNS, including the visual pathway. For example, an ON-patient MRI showed ventricular brain enlargement and atypical thickening of the optic nerve, which were indicative of brain atrophy and optic nerve demyelination [84]. Optical coherence tomography (OCT) and motion perception assessments, both of which examine the visual system, may also be used as adjuncts to MRIs [85]. The majority of neuroimaging techniques may be complex, low reproducibility, uncomfortable and extremely costly (e.g., MRI) [63,79,80]. In contrast, OCT retinal imaging offers a non-invasive, simpler and less expensive technique to provide high-resolution images with detailed analysis of distinct retinal layers [63,79,80,83]. Whilst sophisticated MRI techniques can be used to identify dynamic inflammation, typically seen in earlier stages of MS, standard MRI parameters can be correlated to OCT evidence indicative of irreversible neurodegeneration and, therefore, significant reductions to retinal thickness [79]. Despite the shortcomings of both methods, each provides unique information relating to distinct aspects of MS pathogenesis. This emphasises that both MRI and OCT should be used in conjunction to maximise vital information extraction, enabling in-depth monitoring of MS-associated atypical pathologies and making MS trajectory predictions.

### 3.4. Microglia and Multiple Sclerosis

Like other neurodegenerative disorders, changes in microglial activation has also been observed both in models of MS and in pMS. Experimental autoimmune encephalomyelitis (EAE) is often used in scientific investigations to model MS and ON [48]. Although all experimental models of diseases are not fully representative, studies have shown similarities between human and animal investigations. For instance, compared to healthy, EAE-induced mice were found to have more “de-ramified” amoeboid microglia in the optic nerve and spinal cord [86]. Horstmann et al. found in MS modelled mice by injection of MOG35-55 (encephalitogenic peptide which induces EAE) that retinal microglia were significantly more abundant compared to that of healthy mice injected with PBS [87]. The same investigation also found that, at 60 days post-immunisation, the MS-induced mice had non-significantly but slightly more amoeboid microglia but significantly more ramified microglia compared to the control mice [87]. Another investigation also used MOG35-55 to induce EAE in mice [88]. Within the first week of immunisation, retinal microglia density had increased significantly [88]. In this investigation, amoeboid microglia were also found to be more abundant at 7 and 28 days post-MOG35-55-immunisation; however, no statistical analyses were carried out. These variations of microglial morphotypes indicate that retinal microglia may behave dynamically, becoming “activated” and “de-activated” throughout the progress of EAE. This could correlate to changes in microglial activation, which may occur during fluctuations of demyelination and remyelination seen in MS. Additionally, more sophisticated retinal layer-specific analyses were performed on EAE-induced and healthy mice to find layer-specific microglial morphotypes [86]. Whilst both EAE and healthy mice consisted of ramified microglia in the IPL and OPL, amoeboid microglia were only found in the GCL layer of EAE-induced mice [86]. This interestingly correlates to the GCL and IPL thinning hallmarks of MS, although it has not yet been investigated if this thinning pathology is directly linked to microglial activation. Ultimately, this suggests that retinal microglia may respond and activate in a temporally specific and layer-specific manner [32,86].

Similar implications can also be seen in human studies whereby there was a significantly reduced expression of P2RY12 (homeostatic microglia marker) and significantly increased expression of CD68 in both normal-appearing white matter and areas of active lesions of MS brains compared to healthy brains [89]. Since these markers have been associated with specific functions and microglia morphotypes, the results from Zrzavy et al. suggest that MS brains have fewer surveillant ramified microglia whilst having more phagocytic amoeboid [32,36,89].

Myelin phagocytosis is an additional pathological feature of MS [51]. When Hendrickx et al. investigated the phagocytic capabilities of myelin by microglia through flow cytometry, microglia derived from normal-appearing white matter of post-mortem brains seemed to engulf more myelin from MS donors compared to that from healthy donors [51]. Furthermore, myelin debris processing has been correlated to microglia taking on other “dystrophic” morphologies [51,90]. Cuprizone (CPZ) is a toxic and systemic copper chelator that can be administered intravenously and orally to model MS and demyelinating diseases [90,91]. Cantoni et al. investigated demyelination and microglia characteristics in healthy or CPZ administered [90]. Histological observations revealed that healthy animals were unaffected with their myelin integrity whilst most microglia appeared ramified. Conversely, CPZ administered animals exhibited hippocampal and corpus callosum (CC) neuron demyelination that became more “profoundly” evident with longer exposure to CPZ [90]. Interestingly, amoeboid microglia were abundant up to 4 weeks of CPZ administration, but as this increased to 6 or 12 weeks, more ramified microglia were observed [90]. This suggests that microglial activation plays a more active role in the earlier stages of demyelination or demyelinating diseases such as MS. This is further supported by clinical evidence showing that younger MS patients in earlier stages of the disease are more responsive to therapeutic interventions such as Rituximab that can reduce microglial activation [92,93]. Conversely, transcriptional investigations in microglia from normal-appearing white (NAWM) and grey matter (NAGM) of MS and healthy volunteers showed some interesting differences [94]. Firstly, expression of surveillant microglia markers such as P2RY12 was found to be similar in NAGM and NAWM [94]. Secondly, compared to the NAGM, the NAWM had higher levels of genes involved in the NF-κB pathway, which is involved in the immunomodulatory responses in MS pathology [94]. Unlike the findings from the CPZ MS models [90], van der Poel et al. [94] suggest that surveillant properties of microglia may remain unaffected in the initial stages of MS whilst the transcriptional profile of human-derived microglia are region-specific. Although evidence from human studies would provide a valuable translational comparison, there are, to date, no reports that investigate the morphological characteristics of retinal microglia in patients with MS.

### 3.5. Immunological Markers

Retinal microglia are also unsurprisingly involved in uveitis, an infrequent manifestation of MS. Experimental autoimmune uveitis (EAU) is a commonly used model of uveitis, which can be induced by immunisation of interphotoreceptor retinoid-binding protein (IRBP) [95]. Whole-mounted retinas of EAU-induced mice were found with P2RY12 positive microglia that underwent morphological activation from ramified to amoeboid morphology within 7 days post-IRBP-immunisation, indicating the early involvement of retinal microglia in EAU pathology [95]. Interestingly, ablation of microglia with the antagonist PLX5622 in IRBP-immunised was found to suppressed EAU. This was observed by a decrease in infiltrating inflammatory cells, lack of photoreceptor folds and retinal granulomas compared to untreated animals [95]. Additionally, three-dimensional images were constructed from images of retino-vascular leukocytes, including T-cells and microglia, labelled with CD11b, CD4/CD8, MHCII and P2RY12 [95]. The expression of all markers could be found co-localised with lectin, a marker of blood vessels. Together, these results emphasise the active role of microglia activation in initiating the transport of leukocytes between the blood-retinal-barrier [95]. Whether the morphological shift had a direct correlation to EAU pathogenesis was not explored.

### 3.6. Extracellular Vesicles: Effects on Multiple Sclerosis

The EAE model and MS patients have been investigated in parallel to observe microglia-derived microvesicle (M-MV) EVs in MS/EAE brain pathology [96]. Firstly, M-MVs were found to be neuroinflammatory as evidenced by upregulation of inflammatory markers such as CD86 (T-cell receptor ligand), iNOs and IL1ß, by the transfer of the vesicular components from the origin microglial cell to the recipient [96]. Additionally, EAE mice injected with M-MV into the corpus callosum had many amoeboid microglia, which were not observable in control EAE mice (injected with saline, liposomes or mesenchymal stem cells). Mice with and without acid sphingomyelinase knockout (ASKO) were induced with EAE [96]. ASKO-EAE mice showed significantly reduced M-MVs in the CSF, and CD45^low^CD11b^+^ microglia and inflammatory T-cells in the spinal cord, whilst no ASKO-EAE mice had developed EAE pathologies [96]. In humans, healthy participants were found to have M-MVs in their CSF with increased concentrations in MS: significantly greater in CIS compared to RRMS. These results suggest that M-MVs are differentially present in MS patients and EAE models, which may cause microglial activation and morphotype changes whilst these responses may be reduced by preventing M-MV shedding [96]. This also reveals the potential of targeting M-MVs to monitor disease and its therapeutic strategies. Their role in the retina is still to be established, although if they are found to influence retinal microglia morphology, this could have significant implications for using the eye as a monitoring and treatment efficacy tool.

### 3.7. Genetic Factors: Effects on Retinal Microglia in Multiple Sclerosis

The MOG35-55 induced EAE model of MS was used to identify the genetic and morphological differences of retinal microglia between 8-week-old C57BL/6 mice and healthy controls [97]. Iba-1 staining of cross-sections of the retina revealed mostly ramified microglia in healthy controls whilst there were rounder and amoeboid-like microglia with higher expression of iNOS in early EAE mice (16 days post-MOG35-55 induction—16DPI) [97]. This iNOS expression was reduced by 41DPI, although still higher than that in healthy controls. Additionally, there was increased expression of C1q and TNF-alpha in EAE [97]. This could suggest that early transcriptional changes in retinal microglia reflected by morphological changes may be pathological features of optic neuropathies.

Additionally, Bell et al. investigated the retinal microglia transcriptional profile of Cx3cr1^CreER^:R26-tdTomato C57BL/6 mice that were induced with endotoxin-induced uveitis (EIU) by a single intravitreal injection of LPS [98]. Isolated retinal microglia were analysed with flow cytometry, Fluorescence-Activated Cell Sorting (FACS), mRNA sequencing and quantitative PCR to discover a total of 1069 differentially expressed genes (DEGs) [98]. There were 613 DEGs found at 4 h post-EIU, 537 DEGS at 18 h and none at 2 weeks [98]. Initially, activation markers such as *Fas* and *CD44* were upregulated whilst homeostatic markers such as *P2rY12* and *Mertk* were downregulated in EIU induced samples whilst healthy controls did not show a similar profile, e.g., high P2RY12 levels throughout [98]. Additionally, confocal microscopy of tdTomato^+^ microglial cells in naïve was ramified whilst EIU cells underwent morphological changes from ramified at 4 hours to amoeboid at 18 h, which returned to ramified by 2 weeks post-injection [98]. Together these results indicate that uveitis may be characterised by some initial transcriptional changes that may be reflected by retinal microglia morphology changes [98]. Similar investigations in patient cells would be useful to determine the clinical translatability of these findings.

## 4. Other Neurodegenerative Diseases

### 4.1. Alzheimer’s Disease

AD is the most common cause of dementia. It is characterised by the progressive loss of neurons as a result of the build-up of extracellular amyloid-beta (Aß) plaques and intracellular neurofibrillary tangles composed of hyperphosphorylated tau proteins [99,100,101]. By the time the first cognitive symptoms start to appear, roughly 20 years of irreversible neurodegeneration would already have occurred [100]. Although the exact pathogenesis is still unknown, microglial activation in the brain and retina has been associated with AD pathology [4,13,21,99,100,101,102,103]. This highlights the unmet need for earlier detection of AD pathogenesis and that characterisation of microglia may be a potential answer to this dilemma.

Brain AD investigations have shown more activated microglia in the cortex and hippocampus compared to age-matched controls [99,103]. Specifically, Cao et al. found a significantly greater degree of microglial activation in these areas of AD modelled mice at 12 months old compared to other ages and compared to age-matched or older healthy controls [99]. Additionally, 4-month-old 5× familial AD modelled mice showed that Aß plaques were surrounded by activated microglia in the cortex [103]. These activated microglia were also found to co-localise with Aß aggregates, and as Aß accumulated, microglial cell death was observed [103]. Similarly, Aß plaques were found surrounded by retinal microglia with a large cell body and fewer and thicker ramifications, suggesting activated microglia observations could be seen in the AD modelled retina [100,101,102]. Some sources have found activated retinal microglia in 3× transgenic AD mice as young as 5 postnatal weeks old, suggesting an initial microglial response in AD pathology [102]. This suggests that some microglial responses to AD may occur in the retina before the brain. Despite these findings, it is important to note the ambiguous definition of activated microglia. For instance, some literature refers to all non-resting microglia as “activated microglia”, and some may use the term interchangeably with “activation” of microglia, whilst more recent literature may use the term in reference to the unique morphological description of the activated microglia.

Rod microglia may have a region-specific involvement in AD pathology [4,21]. For instance, when three brain regions of AD patient autopsies were examined, rod microglia were only seen in the parietal cortex and not in the hippocampus or temporal cortex [4]. Additionally, there were no significant observations seen relating rod microglia in hippocampal and cortical brain regions of patients who had traumatic brain injury [4]. However, rod microglia were found to be significantly abundant in the parietal cortex but not the hippocampus of AD patients compared to healthy controls [4]. Rod microglia have also been observed in those with mutated *C9orf72* whilst they were absent in age-matched controls whilst additionally, a high density of rod microglia in the grey matter of those with Down’s Syndrome AD correlated to more severe pathological hallmarks compared to those just with AD [21].

Recently, Grimaldi et al. investigated the retinal pathology in post-mortem AD patient retinas [15]. These samples also had Aß plaques and phosphorylated tau tangles, with signs of retino-neuronal degeneration evidenced by increased expression of caspase 3, a marker of AD-associated neurodegeneration [15]. Additionally, the AD retinal slices examined had a significantly higher density of iba-1 positive microglial cells which also expressed higher levels of DAM marker, IL-1ß, compared to healthy samples [15]. TREM2 is also a DAM associated marker, although this was not upregulated in the iba-1 positive AD microglia [15]. Despite this, the AD retinas expressed higher levels of TREM2 mRNA compared to healthy controls [15]. This unexpected trend in AD retinal microglia may be due to historic evidence of post-mortem retinal samples with Aß deposits expressing downregulated TREM2 [15]. Investigations that observe other retinal DAM markers in parallel to morphological parameters may provide useful insight to elucidating AD retino-pathologies.

### 4.2. Parkinson’s Disease

Parkinson’s disease (PD) is the second most common type of neurodegenerative disease, affecting over 10 million people worldwide [104,105]. Its pathological hallmark consists of Lewy body (LB) aggregates composed of alpha-synuclein (α-syn) that accumulate inside the neuronal cell bodies and axons [104,105]. This is followed by the gradual neurodegeneration of dopaminergic neurons in CNS regions such as the substantia niagra (SN) pars compacta [6,106,107]. By the time the initial clinical symptoms such as tremor, rigidity, akinesia and postural stability start to appear, 50–80% of dopaminergic neurons have already been lost [107,108]. Much like AD, PD research has revealed many potential mechanisms of pathogenesis, including neuroinflammation as a result of microglial activation [6,105,109]. Recent studies have also revealed that up to 80% of PD patients may experience some visual dysfunction at least once throughout their disease progression [105]. Additionally, some studies have reported these retinal changes to occur prior to that of the brain [110]. Considering these factors, exploring the retinal microglia characteristics may serve as an ideal starting point in identifying earlier biomarkers of PD.

One study investigated the characteristics of SN and hippocampal microglia in three participant groups: PD patients, matched healthy participants and prodromal PD (p-PD) participants, with age-matched analyses performed (age range from 56 to 96 years old) [111]. Whilst there were almost no α-syn and LBs found in both brain regions of healthy controls, there were some in that of the p-PD patient specimens and significantly more in PD [111]. Additionally, healthy controls had significantly more ramified microglia compared to p-PD and PD groups, p-PD had significantly more amoeboid microglia compared to healthy participants, and PD patients also had significantly more than p-PD [111]. “Primed” or activated microglia were prominently seen in p-PD compared to any other participant group. This suggests that activated microglia may be an early indicator whilst amoeboid microglia are associated with PD pathology.

Similarly, healthy retinas were free of α-syn whilst there were some in 5-month-old retinas of transgenic mice (TgM83) modelled for PD and significantly more in the OPL of 8-month-old TgM83 retinal samples [112]. Only 8-month-old mouse retinas indicated microglial activation due to significant increases of CD68 expression (microglial activation marker) and visual observation of amoeboid microglia, whilst this was not seen in other experimental groups [112]. This suggests that there may be a differential expression of activated microglial markers and morphological changes through the early to intermediate stages of PD pathology. However, there are other studies that show that whilst there was OPL α-syn accumulation in mice modelled for α-syn-dependent diseases (Plp-α-syn), there were mostly ramified microglia found in whole-mounted retinal samples with no sign of microglial activation for all age groups investigated [113]. This may have been due to differences in disease models used, age groups investigated, and methods of analysis.

### 4.3. Glaucoma

Glaucoma is one of the main causes of blindness worldwide, which is estimated to affect more than 120 million people by 2040 [114]. It is characterised by the gradual loss of RGCs and, like AD and PD, significant neurodegeneration would have already occurred before clinical symptoms start to emerge [114]. Numerous methods for detecting the activity of glaucoma have been developed, including elevated intraocular pressure and an increased numbers of dying retinal cells, which is measured by detecting apoptosing retinal cells (DARC) [115,116]. Additionally, microglial activation may also play a role in glaucoma pathogenesis.

Microglia activation has been identified as one of the first events in glaucomatous neural damage, which may occur before RGC death [117,118]. For instance, when Swiss albino mice were modelled with the unilateral ocular hypertension (OHT) model of glaucoma, there was microglial activation [108]. This was seen in the form of increased cell body size and retraction of cell processes by as early as 1 day post-surgery (PS) and increased cell density by 3 days PS. Ramírez et al. additionally found that retinal microglia were P2RY12+ at 1 day PS [108]. This expression was decreased at 3 and 5 days PS, which then returned to normal levels by 15 days PS [108]. Moreover, the extent of neurodegeneration could also be correlated with early microglial changes, including microgliosis or microglial activation, as evidenced in transgenic *Cx3cr1*^GFP/+^ DBA/2J mouse retinas modelled for pigmentary glaucoma [117]. In addition, in another experimental glaucoma model, treatment with minocycline or with a high dose of irradiation reduced microglial activation, resulting in lower RGC death [119,120].

Rod microglia have also been associated with several disease states. In a model of progressive RGC degeneration in rats, it has been shown that optic nerve transection (ONT) induced retinal rod microglia formation [23]. This was observable within the first 7 days, peaking at 14 days and disappearing after 2 months [23]. These periodic trends were interestingly reflected by the trends of ONT-induced RGC death. Additionally, these retinal rod microglia were found to orient themselves along the axon and cell body of RGCs, suggesting their active role in ONT-associated neurodegeneration [23].

### 4.4. Inherited Eye Disease: Retinitis Pigmentosa

Another major cause of inherited irreversible blindness is retinitis pigmentosa (RP). It affects roughly 1 in 4000 people in the world. Typically, patients may undergo loss of retinal rod cells, whilst more advanced cases may be caused by loss of cone cells. Although the exact mechanism of RP is still unclear, there is evidence of early retinal microglia activation occurring initially in the process of photoreceptor degeneration [121]. For instance, Di Pierdominico et al. observed P23H-1 rats, a model of inherited photoreceptor degeneration [121]. A reduction in rows of photoreceptor nuclei occurred between P10-21 with a greater reduction between P15-21 [121]. This occurred simultaneously with microglial morphology changes to amoeboid microglia, observed through retinal cross-section histology analyses showing that iba-1 positive microglia with shorter processes by P15 whilst the total retinal microglia density was significantly greater in P23H-1 rats compared to controls between P15-45 [121]. The same study investigated Royal College of Surgeons (RCS) rats, another model for inherited photoreceptor degeneration, to find a similar trend. The most significant photoreceptor degeneration occurred between P33-60 whilst retinal microglia took on the amoeboid appearance by P21-33 [121]. These microglia also migrated to the outer retinal layers between P21-60. RCS retinas had a significantly higher density of microglia compared to controls P21-60, whilst there was a significant decline between P45-60 [121].

## 5. Perivascular Microglia

Investigating microglia that reside in or around the perivascular region (PM) has become a popular field of research because of the associations that have been made between immune responses to neurological conditions and the CNS vascular network [122]. These associations are seen where patients with diabetic retinopathy had a significantly elevated number of hyper-ramified retinal PM microglia cells in comparison to that of normal subjects [34]. Additionally, PM microglia are said to closely track any incoming compounds from outside the blood-brain barrier through the vessels and into the CNS [95]. For instance, drug-induced depletion of PM microglia in mice with experimental AD resulted in corticovascular accumulation of amyloid plaques [122]. Other investigations also found that glaucoma modelled mice (ocular hypertension model induced by laser) presented with rod microglia both in the retinal PM and adjacent to the retinal axons [22]. This indicates that microglia morphotypes are not specific to perivascular regions.

## 6. Imaging Retinal Microglia

The establishment of a robust method to image retinal microglia both non-invasively and in vivo would provide an invaluable avenue to tracking disease-associated retinal microglia pathologies. There are indeed transgenic, e.g., CX3CR-1^GFP^ mice, which are specially bred to have enhanced expression of the green fluorescent protein (GFP) in the chemokine receptor 1 (CX3CR-1) gene [123,124,125]. As a result, scanning laser ophthalmoscopes (SLOs) and OCT systems may be used to non-invasively image the fluorescing retinal microglia at a cellular resolution, to investigate their shape, spatial distribution and density in real time [37,124,125,126]. Each of these studies could use distinctive methods with unique SLO/OCT systems including: multi-colour confocal SLO to detect multiple fluorescent markers [124], widefield autofluorescence imaging [125] and 488 nm fluorescence imaging using commercial SLO/OCT devices [126]. Although this reveals the imaging versatility of the Cx3CR-1^GFP^ mice, genetic modifications often have undesirable adverse effects and are not currently clinically translatable.

Despite this, there have also been reports of attempts of in vivo and non-invasive imaging methods in humans [127,128]. For example, Liu et al. developed a multimodal adaptive optics (AO) system which combined SLO and OCT systems [127]. Superluminescent diodes were also used to produce the imaging beams from each of the AOSLO and AOOCT with the addition of mirrors positioned precisely for it to be directed into the eye, enabling the retina to be imaged. Although they were able to image microglia in the inner limiting membrane (ILM), these were identified by subjective visual interpretation, and the authors emphasise the need for improvement due to the lengthy process (1 hour to image) [127]. Although AOOCTs can produce high-resolution and cellular levels of imaging, this is compromised by ~6-fold reduction in the field of view compared to a commercial machine [128]. For instance, Castanos et al. used spectral-domain en face OCT imaging in the reflectance mode (OCT-R) to obtain larger images of the retina [128]. On examination of the ILM in healthy participants, uniformly distributed ramified macrophage-like cells were observable [128]. In patients with diabetic retinopathy, central retinal vein occlusion and open-angle glaucoma, these cells appeared amoeboid and not uniform [128]. Through these morphological and distributional characteristics, the authors interpret these cells as microglia or hyalocytes. Although this reveals the possibility of non-invasive in vivo imaging of the human retina, the authors recognise limitations to their technology, including manual subjective interpretations of cell morphology and the indefinite cell type distinction [128].

## 7. Contradictory Findings: Reasons and Limitations

Although the aforementioned studies had similar objectives of finding densitometric or morphological features of microglia, there were clearly some which produced contradictory results. This may have been because many of the methods of analyses used were subjective, varied and even ambiguous. These aspects could have been amended to allow for more consistent, representative and less biased results. For example, when specific regions of the CNS, such as the striatum, SN, hippocampus or the retina, have been investigated, randomly chosen “sections” or “slices” are analysed [86,111,129]. Additionally, some investigations took subjective measures to distinguish “ramified” and “amoeboid” cells whilst also using manual counting methods [99]. There are indeed other methods that can certainly be used to reduce the impact of these subjective and non-representative techniques. For instance, more extensive and efficient imaging methods can be achieved by scanning the whole retina rather than sectors and including z-stacks of all the layers and maximum intensity projections [24]. In this way, observations of the whole retina and all its layers can be comprehensively analysed. Furthermore, algorithms that automate cell counting can be used to quantify densitometric parameters [24]. In light of encouraging future investigations, avoidance of subjective categorisation of microglia morphotypes has successfully already been performed using changes in 15 parameters defined through the course of microglial inflammation enabling morphometric analyses output by automated software (Fraclac; ImageJ) [130]. However, it is important to note that Fernández-Arjona et al., manually selected the microglia cells to be analysed based on a set of criterion which consisted of: fully visible cell body and processes which are nonoverlapping with parts of other adjacent cells [130]. Therefore, the resulting data were not fully representative or free from bias, although this clearly demonstrates the principle of using quantitative data to determine the different morphotypes.

Clinical and preclinical trials often have only male subjects [60,91]. This may be in order to reduce the effects of physiological differences such as the ovarian cycle, confounding the results of the trial or experiment. However, are known sexual dimorphisms for the structural and functional differences in microglial responses to ageing and disease, including MS [131]. This may be due to X chromosomes that express the greatest number of immune-related genes in the whole human genome and epigenetic and environmental influences [131,132]. Han et al. also suggest that hormonal differences may influence males having more activated microglia in development stages, putting them at higher risk of disease, whilst for females, this occurs during adulthood [131]. Other investigations on mediating hypersensitivities to pain have shown the involvement of microglia in male mice whilst other immune cells such as T-cells are required in female mice [133,134]. Although there are no reports which investigate the sexual differences in the morphological characteristics of retinal microglia in MS, transcriptome microarray analyses of male and female mice of two age groups (3 or 24 months) have shown that age-associated changes of retinal microglia cell-specific gene expression are sexually dimorphic [135]. Designing trial/experimental studies based on one sex may limit the ability to identify sex-associated pathological differences in MS, a disease more common in females [60]. Using stratified analyses in investigations that are inclusive of both sexes instead would benefit understanding pathological differences between different sub-sets of patients within the heterogeneous MS population. This would also enable larger patient group sizes to be investigated in larger multicentre research collaborations with consequently greater impact and generalisability.

There has also been a recent interest in the use of computer vision and machine learning algorithms to enable automated recognition and/or categorisation of “cells” or “regions of interests” in neuroimaging data. Commonly used in biomedical research are support vector machines (SVMs), regarded as supervised machine learning techniques that can categorise data and perform regression analyses [136]. Linear SVMs can use the input “training” data for each category to extract features that may be used to define each category [137]. Then, it uses the input “training” data to effectively categorise each data point by examining said defining features [137]. This is achieved by automatically identifying the optimal hyperplane that allows each data point to be distinguished in one category from those of another. These machine learning algorithms have already been implemented in research in efforts to predict dementia in patients using data from high-resolution MRIs and even mental state assessment scores [136]. Although these studies do not yet produce algorithms that have 100% accuracy and precision, the concept of using shareable automated algorithms presents as an excellent starting point to improving biomedical data processing and analysis [136].

## 8. Concluding remarks

In conclusion, microglia offer an exciting avenue for research in neurodegenerative disorders, including MS. There is also mounting evidence to show some early retinal microglial changes in response to diseases, further supporting the idea of using retinal biomarkers as an earlier method of disease detection. Additionally, there is a growing number of studies that investigate retinal and brain microglia with respect to its morphological, genetic and molecular characteristics. However, the key to our understanding of their role is the ability to perform accurate and consistent quantitative analyses of the dynamic changes in structure, as morphotypes may be closely linked to microglia function. We would encourage the development of standardised experimental designs and validated data analysis algorithms, as these are necessary to comprehensively delineate the role that these important cells have in disease pathogenesis and severity.
